# Supplementary material for: An open-source framework for large-scale, flexible evaluation of biomedical text mining systems
Source: J Biomed Discov Collab. 2008 Jan 29;3:1. doi: 10.1186/1747-5333-3-1 (PMC2276192; doi:10.1186/1747-5333-3-1)
Supplement: Additional file 1 — Evaluation platform source code. The additional file contains the software evaluation framework discussed in this paper. The most current release can be downloaded from SourceForge.net [1]. See the accompanying README file for instructions on installation and use. Also included in the distribution is a collection of UIMA wrappers for some commonly used BioNLP tools and annotated corpora. [file 1747-5333-3-1-S1.zip › bionlp_uima_repository-1.0.1/docs/api/src-html/edu/uchsc/ccp/util/nlp/mention/comparison/IdenticalComplexSlotMentionComparator.html]

```
001    package edu.uchsc.ccp.util.nlp.mention.comparison;
002    
003    import edu.uchsc.ccp.util.nlp.annotation.comparison.SpanComparator;
004    import edu.uchsc.ccp.util.nlp.mention.ClassMention;
005    import edu.uchsc.ccp.util.nlp.mention.ComplexSlotMention;
006    import edu.uchsc.ccp.util.nlp.mention.Mention;
007    
008    public class IdenticalComplexSlotMentionComparator extends IdenticalMentionComparator implements ComplexSlotMentionComparator {
009    
010        /**
011         * Compares two mentions. They must be ClassMentions or else an UnsupportedOperationException is thrown. The CSMs must have identical mention
012         * names, and identical ClassMentions fillers.
013         * 
014         */
015        @Override
016        public int compare(Mention mention1, Mention mention2, SpanComparator spanComparator, int maximumComparisonDepth, int depth) {
017            if ((mention1 instanceof ComplexSlotMention) & (mention2 instanceof ComplexSlotMention)) {
018                return compare((ComplexSlotMention) mention1, (ComplexSlotMention) mention2, spanComparator, maximumComparisonDepth, depth);
019            } else {
020                throw new ClassCastException("Cannot support comparison between a SlotMention and a non-SlotMention");
021            }
022        }
023    
024        /**
025         * Compares two complex slot mentions. The CSMs must have identical mention names, and identical ClassMentions fillers.
026         * 
027         * @param complexSlotMention1
028         * @param complexSlotMention2
029         * @param maximumComparisonDepth
030         * @param depth
031         * @param spanComparator
032         * @param compareLinkedAnnotationSpans
033         * @return
034         */
035        public int compare(ComplexSlotMention complexSlotMention1, ComplexSlotMention complexSlotMention2, SpanComparator spanComparator,
036                int maximumComparisonDepth, int depth) {
037            if (complexSlotMention1.getClassMentions().size() == complexSlotMention2.getClassMentions().size()) {
038                /* if the cms's do not have an equal number of class mentions then return false */
039                if (hasEquivalentMentionNames(complexSlotMention1, complexSlotMention2)) {
040                    /* initialize a new IdenticalClassMentionComparator to compare the ClassMentions contained in each CSM */
041                    IdenticalClassMentionComparator icc = new IdenticalClassMentionComparator();
042                    for (ClassMention cm : complexSlotMention1.getClassMentions()) {
043                        boolean cmHasMatch = false;
044                        for (ClassMention cmToCompare : complexSlotMention2.getClassMentions()) {
045                            if (icc.compare(cm, cmToCompare, spanComparator, maximumComparisonDepth, depth) == 0) {
046                                cmHasMatch = true;
047                            }
048                        }
049                        if (!cmHasMatch) {
050                            return complexSlotMention1.getSingleLineRepresentation().compareTo(
051                                    complexSlotMention2.getSingleLineRepresentation())
052                                    * MULTIPLIER;
053                        }
054                    }
055                    /* if we get to this point, then all class mentions have a match, so return 0 */
056                    return 0;
057                } else {
058                    /* the csm's have different mention names */
059                    return complexSlotMention1.getSingleLineRepresentation().compareTo(complexSlotMention2.getSingleLineRepresentation())
060                            * MULTIPLIER;
061                }
062            } else {
063                /* the csm's have an unequal number of class mentions */
064                return complexSlotMention1.getSingleLineRepresentation().compareTo(complexSlotMention2.getSingleLineRepresentation())
065                        * MULTIPLIER;
066            }
067        }
068    
069    }
```
